# Real time contrast enhanced ultrasonography in detection of liver metastases from gastrointestinal cancer

**DOI:** 10.1186/1471-2407-7-171

**Published:** 2007-09-03

**Authors:** Fabio Piscaglia, Francesco Corradi, Mikaela Mancini, Francesco Giangregorio, Stefano Tamberi, Giampaolo Ugolini, Bruno Cola, Alberto Bazzocchi, Roberto Righini, Patrizia Pini, Fabio Fornari, Luigi Bolondi

**Affiliations:** 1 Div. Internal Medicine, Azienda Ospedaliero-Universitaria S. Orsola-Malpighi, Bologna, Italy; 2 Div. Gastroenterology, Ospedale Pietro da Saliceto, Piacenza, Italy; 3 Div. Oncology, Ospedale Civile di Faenza, Italy; 4 Div. Emergency Surgery, Azienda Ospedaliero-Universitaria S. Orsola-Malpighi, Bologna, Italy; 5 Div. General Surgery, Azienda Ospedaliero-Universitaria S. Orsola-Malpighi, Bologna, Italy

## Abstract

**Background:**

Contrast enhanced ultrasound (CEUS) is an imaging technique which appeared on the market around the year 2000 and proposed for the detection of liver metastases in gastrointestinal cancer patients, a setting in which accurate staging plays a significant role in the choice of treatment.

**Methods:**

A total of 109 patients with colorectal (n = 92) or gastric cancer prospectively underwent computed tomography (CT) scan and conventional US evaluation followed by real time CEUS. A diagnosis of metastases was made by CT or, for lesions not visibile at CT, the diagnosis was achieved by histopathology or by a malignant behavior during follow-up.

**Results:**

Of 109 patients, 65 were found to have metastases at presentation. CEUS improved sensitivity in metastatic livers from 76.9% of patients (US) to 95.4% (*p *<*0.01*), while CT scan reached 90.8% (p = n.s. vs CEUS, p < 0.01 vs US). CEUS and CT were more sensitive than US also for detection of single lesions (87 with US, 122 with CEUS, 113 with CT). In 15 patients (13.8%), CEUS revealed more metastases than CT, while CT revealed more metastases than CEUS in 9 patients (8.2%) (p = n.s.).

**Conclusion:**

CEUS is more sensitive than conventional US in the detection of liver metastases and could be usefully employed in the staging of patients with gastrointestinal cancer. Findings at CEUS and CT appear to be complementary in achieving maximum sensitivity.

## Background

The presence of liver metastases is an important determinant of survival in colorectal cancer patients [1-4], accounting for >50.000 deaths per year in the United States alone [2]. Prognosis is inversely related not only to the presence of metastases but also the number and volume [3,4]. In recent years, it has become accepted that hepatic metastasectomy, possibly repeated in cases of recurrence, prolongs survival in comparison to even the best medical treatment; therefore, surgery should be offered to all patients not presenting extrahepatic spread, who, depending upon co-morbidities and extent of the parenchyma to be sacrified, are elegible for this procedure [5,6]. Accurate and timely detection of hepatic metastases, both at diagnosis and during follow-up, is, therefore, very important on account of the critical therapeutic and prognostic implications. Accurate assessment of the number, size and site of metastases is mandatory to identify those patients elegible for surgical treatment, or to forecast prognosis in the event of other treatment modalities for unresectable cases.

Pre-operative imaging techniques may fail to detect small metastases i.e., <1–2 cm in size [7-11] which, when below 1 cm, may be overlooked, even at liver inspection and palpation during surgery [7,9-13]. Of the various techniques available in the detection of hepatic metastases, conventional ultrasound (US) is slightly less sensitive than Computed Tomography (CT) and significantly less sensitive than magnetic resonance imaging (MRI) and Positron Emission Tomography (PET) [14-21]. However, despite these limitations, US is usually the first-line investigation in the assessment of patients with gastrointestinal cancer in Italy, due to its low cost, non-invasiveness, repeatability and easy access. MRI and PET, and often CT, are, at least in Italy, restricted to selected cases, due to their limited availability and high costs. Thus, the introduction of new techniques to increase the sensitivity of US in the detection of metastases would be a major advantage.

In 2002, new grey scale US techniques based on the harmonic response to second generation contrast agents, insonated with low acoustic pressure (low mechanical index), became available. These techniques employ contrast microbubbles resonance and, avoiding contrast destruction, allow real-time exploration of liver perfusion [22].

As in MRI and CT, the use of contrast agents appeared to enhance the sensitivity of US in the detection of liver metastases, regardless of the primary tumor [11,14,16-18,23]. So far, no study, to our knowledge, was carried out to investigate the use of real time contrast-enhanced US (CEUS) for liver staging selectively in gastrointestinal cancer patients.

Aim of the present investigation was to compare the accuracy of real-time CEUS, in the detection of hepatic metastases from colorectal and gastric cancers, with that of two commonly employed imaging techniques, namely conventional B-mode US and CT.

## Methods

A total of 120 patients, with recently diagnosed colorectal or gastric cancer, referred to the two study Units for abdominal US, were prospectively taken into consideration for enrolment in the investigation. Patients were consecutive, as observed at both the US study Units. Exclusion criteria were the finding of >4 metastases at conventional US or controindication to CT. This limit in the number of metastatic lesions was set in order to select patients in whom a more accurate liver staging could, theoretically, modify the treatment strategy. A total of 109 patients (64 males, 45 females, mean age 66 years, range 39–85) were finally enrolled in the study.

The reference method for the diagnosis of metastatic lesions was the typical appearance at CT. Lesions detected only by US techniques or with an inconclusive CT appearance had to be confirmed as being of metastatic nature by inspection and/or pathological findings in the case of surgical resection, or by percutaneous biopsy or by means of multimodality imaging at follow-up (increase in size and/or clear signs of a malignant nature, such as infiltration of surrounding structures or progressive decrease in size/disappearance under chemotherapy).

**Figure 1:**
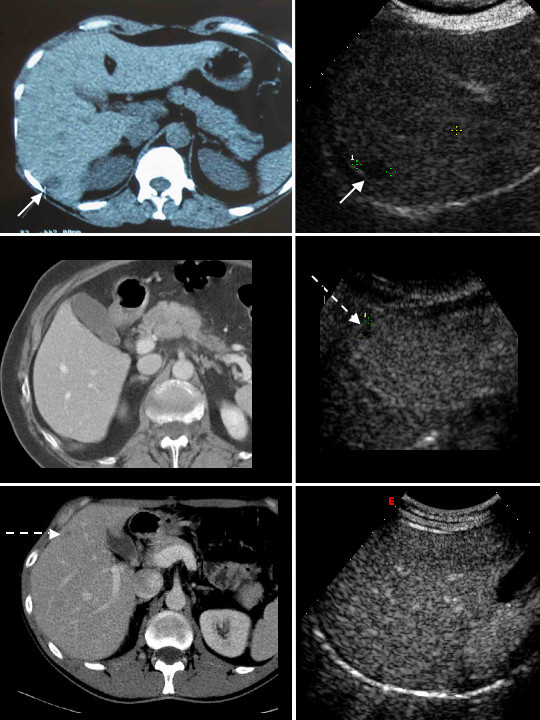
Comparison of CT (left column) and contrast enhanced US (CEUS) (right column) appearance of liver metastases in the venous-late phases of portal perfusion. In the upper row a concordance is shown in the detections of a subcapsular metatasis localized in liver segment 7. Metastasis appears hypodense at CT and hypoechoic at CEUS (continuous-line arrows). In the middle and lower rows, two cases of discordance between CT and CEUS are shown. In the middle row the tiny subcapsular metastasis (dashed-line arrow) was detected by US, but not by CT. The metastastic nature of the lesion was confirmed intraoperatively at the time of resection of the primary cancer plus metastasectomy. In the lower row, the metastatic lesion (dashed-line arrow), located anteriorly in segment 6, was identified by CT and missed by CEUS. The malignant nature of the lesion was confirmed at surgery.

**Figure 2:**
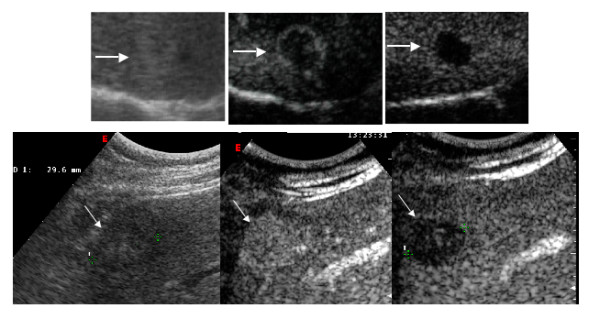
Patterns of liver metastases at CEUS. Lesions are indicted by white arrows. Upper row. In conventional B-mode ultrasound (left frame) the focal liver lesions is hardly visibile, showing a faint hyperechoic apperance. During the arterial phase (23 seconds after contrast injection, central frame) the lesion shows a rim- like hyperechoic aspect, whereas it becomes practically anechoic during the late phase (160 seconds after injections, right frame), consistently with a metastasis from left colonic adenocarcinoma. Lower row: in conventional B-mode ultrasound (left frame) this metastasis from rectal adenocarcinoma appears inhomogeneous, predominantly hypoechoic, whereas it becomes homogeneously hyperechoic during the arterial phase (32 seconds after contrast injection, central frame) and washes contrast out in the late phase, becoming markedly hypoechoic (125 seconds after injection, right frame).

The site of the primary tumor was colon in 86 patients, rectum in 6 and stomach in 17. Patients were enrolled over a 24-months period and were observed at follow-up for at least 6 months after CEUS examination; written informed consent to enter the study was obtained from all participants. CEUS studies were performed using commercialy available US systems (Technos MPX^® ^or Megas GPX^®^, Esaote™, Genova, Italy) and 3.5-MHz transducers, as described elsewhere [24]. US of the liver was first performed with conventional B-mode. Metastases were identified according to conventional imaging criteria. Thereafter, the contrast investigation was carried out following i.v. infusion of the US contrast agent in a cubital vein. All patients received a bolus infusion of Sonovue^® ^(Bracco™, Milan, Italy); patients >70 kg received 4.8 ml of contrast agent. Slim patients (<70 kg) in whom perfect liver US explorability was possiblle, received 2.4 ml of contrast. For all other patients <70 kg the choice between 2.4 and 4.8 ml of SonoVue was left to the operator, the aim being to obtain all relevant diagnostic information with the least amount of contrast, in order to reduce costs. All contrast infusions were immediately followed by a 5 ml saline flush to clear the infusion line and to prevent a decrease in the contrast agent flow in the arm veins. CEUS was performed using contrast specific imaging software at low acoustic power output (Mechanical Index < 0.1) (CnTI^® ^Esaote™, Genova, Italy), by one operator in Piacenza (F.G.) and by one of two operators in Bologna (F.P., M.M.). The same specific contrast detection imaging software was present in both types of equipments and employed throughout the study. The liver was assessed, in all patients, in the arterial (10–35 sec), portal-venous (40–120 sec) and late phases (>120 sec) following injection of SonoVue, according to published guidelines [22]. The liver was explored until a marked overall decrease in contrast signal intensity occurred or complete disappearance of contrast was reached, which, on average, occur 3–6 minutes after contrast injection. Metastatic lesions were considered those solid lesions showing contrast wash-out in the portal phase, thus becoming markedly hypoechoic or even anechoic in the late phase (Fig. 1, 2), regardless of the type of arterial phase pattern, in accordance with accepted criteria [22]. The number, site and size of metastases, at baseline and contrast US, were recorded at the time of US examination.

All patients also underwent contrast-enhanced CT, either with 4-detector multislice CT (approximately two thirds of the patients) or single-detector helical CT (the remaining patients) performed within 4 weeks of the US examination (either before or after). All CT scans were performed in the Radiology Units used by the referring physician. The number, site and size of metastases were recorded at the time of CT reporting.

US operators were blind to CT findings and viceversa, whereas CEUS readers were fully aware of the findings of conventional US exams, since the latter were carried out immediately before CEUS examinations and by the same operator.

The segmental distribution of the lesions was recorded in all 3 modes. The agreement in lesion location was obviously consistent in conventional US and CEUS mode, since the same operator performed both techniques, the imaging approach was the same and the techniques were performed practically at the same time. When, on the other hand, a discrepancy was recorded in the location of a lesion, between CT and US mode(s), the CT films and US images/clips were retrospectively reviewed to assess whether the same lesion was visualized by the various modalities, but described in different segments or whether the two imaging modalities had visualized two distinct lesions.

The prevalence of benign lesions (e.g. hemangiomas, simple biliary cysts) was not within the scope of the present study and is not reported herein.

The study was carried out in accordance with the Helsinki declaration and was approved by the Ethics Committees of the S. Orsola-Malpighi hospital in Bologna and Pietro da Saliceto hospital, Piacenza, Italy, corresponding to the two centers where CEUS was performed.

### Statistical Analysis

Differences in the diagnostic accuracy of the various imaging modalities were investigated in connection with: 1) the rate of cases with metastatic disease; 2) the total number of metastatic lesions detected. Analysis of variance (ANOVA) was performed to compare CT, conventional B-mode and CEUS findings. Sensitivity in the detection of liver metastatic cases, by the three imaging modalities, was calculated on a patient basis and compared using McNemar's test. In the detection analysis, a Wilcoxon's matched pairs signed rank test was used to test the difference in the number of lesions found by the two imaging modalities. A "p" value < 0.05 was considered statistically significant.

## Results

### Accuracy in identifying patients presenting metastatic liver disease

Of the 109 patients, 65 presented liver metastases and 44 did not. CEUS enhanced the sensitivity in the detection of metastatic patients from 76.9% (B-mode) to 95.4% (CEUS) (p < 0.001), while CT-scan reached 90.8% (p = 0.035 vs B-mode) (Table 1). A total of 47 patients were correctly classified as metastatic by all 3 imaging techniques. Of the remaining 18 metastatic patients, 9 (8.3% of all patients) were diagnosed as metastatic both by CEUS and CT, but not by B-mode US; CEUS revealed metastatic disease in 6 patients (5.5%), of whom 3 detected also by B-mode conventional US, but not revealed as metastatic by CT (Table 2); CT revealed metastatic disease in 3 patients (2.8%) who, instead, had resulted negative at CEUS (Table 2) and at baseline US (Table 2).

**Table 1:** Sensitivity of imaging in detection of metastases

**Imaging modality**	**Cases (Sensitivity%)**
CEUS	62 (95.4%)
Conventional b-mode US	50 (76.9%)
Spiral CT	59 (90.8%)

Value of p calculated using Mc Nemar Test:

CEUS *vs *CT: *p *= *0.508*

CEUS *vs *Conventional b-mode US: *p *<*0.001*

CEUS *vs *CT: *p *= *0.035*

CEUS, contrast enhanced ultrasonography; CT, computed tomography. Total number of case-patients with liver metastases = 65.

**Table 2:** Identification of patients with liver metastases using various imaging techniques

	**Dual-Phase CT**	***CEUS***	**Conventional b-mode Ultrasound**	**Reference method for discrepancies**
**Patients *(n)***^**1**^	- *(44)*	- *(44)*	- *(44)*	
**Patients *(n)***^**1**^	+ *(47)*	+ *(47)*	+ *(47)*	
				
**Patients *(n)***^**2**^	+ *(9)*	+ *(9)*	- *(9)*	
	+ *	-	-	*Surgery*
	+ *	-	-	*Surgery*
	-	+ §	*+ *#	*Surgery*
	-	+ *§*	*+ #*	*Biopsy*
	-	+ *§*	*+ #*	*MRI*
	-	+ *§*	-	*MRI*
	-	+ *§*	-	*F-up*
	-	+ *§*	-	*F-up*
	+ *	-	-	*F-up*

CEUS, contrast enhanced ultrasonography; CT = computed tomography.

^1 ^A total of 44 patients were negative (-) for metastases with all imaging techniques, while 47 were positive (+) showing the same lesions with all techniques (first two lines). Remaining lines refer to patients in whom imaging findings showed a discrepancy in classifying them as metastatic.

^2 ^in 9 patients CT and CEUS were concordant, being more sensitive than conventional US. In the remaining other 9 patients a discordance was reported among the three techniques. Each line provides information about a single patient. Since in these 9 patients, CT and CEUS findings were not consistent, confirmation of the results was obtained by a further reference modality, as specified in the right column (MRI = Magnetic Resonance Imaging, F-up = frank progressive metastatic pattern during follow-up, Surgery = intraoperative or pathologic confirmation at the time of laparotomy for metastasis resection).

* patients with liver metastasis at CT, but negative with other techniques.

§ patients positive at CEUS and negative at CT.

# patients positive at both conventional US and CEUS and negative at CT.

A few patients (<8% of total) showed suboptimal or poor explorability of the liver at US imaging modalities according to the US operator's subjective judgement. The cause was either liver location (relatively high) and/or liver echogenicity (bright/fatty liver). Two of these patients belonged to the cases classified as false at CEUS. Indeed, the negative predictive value for metastastic disease was 77.2% for baseline US, 88.0% for CT and 93.6% for CEUS

### Identification of individual metastases and comparison between the various imaging techniques

CEUS significantly enhanced also the sensitivity in the number of metastatic lesions detected in comparison to conventional US. Baseline US revealed 87 metastases, while CEUS identified 122 metastases and CT-scan 113 (Table 3). The total number of metastases detected was 132. Of these 132 lesions, the diagnosis was reached, according to CT as reference method, in 113. The 19 lesions detected by CEUS, but not by CT were diagnosed as follows: 7 intraoperatively, 1 by liver biopsy and 11 by increase in size during follow-up (a few following an initial decrease under chemotherapy). The smallest lesion identified by CT was 8 mm in maximal diameter and 7 mm the smallest by CEUS and US.

**Table 3:** Number of patients classified by each imaging technique, according to number of metastatic lesions detected (left column). Total of 65 patients presented metastases

**Liver Metastases *(n)***	**Dual-Phase CT**	**CEUS**	**Conventional b-mode Ultrasound**^**1**^
0	6	3	15
1	29	30	27
2	15	15	12
3	11	10	8
4	2	5	3
5	-	-	-
6	1	2	-
7	1	-	-

CEUS, contrast enhanced ultrasonography; CT, computed tomography.

^1 ^For conventional b-mode US ≤ 4 lesions were allowed according to inclusion criteria.

#### Comparison between CEUS and conventional B-mode US

In 39 of the 65 metastatic patients (60%), the number of focal metastatic lesions detected at CEUS and at conventional B-mode US was identical. An increase was observed in the number of detected lesions, on CEUS mode, compared with conventional B-mode US in 26 out of the 65 patients. The mean number of lesions per metastatic patient increased from 1.34 to 1.88 (p < 0.001 Wilcoxon's matched pairs signed rank test). Conventional B mode US did not reveal more lesions than CEUS in any of our patients.

#### Comparison between CEUS and dual-phase CT

In 41 of the 65 metastatic patients (63%), the number of lesions observed at CEUS and at dual-phase CT was identical. In the remaining 24 patients (37%), the number of lesions varied. In 15/65 (23.1%) cases, 19 lesions were seen at CEUS, but not at dual-phase CT, whereas in 9/65 (13.9%) patients, 10 lesions were seen at dual-phase CT and missed at CEUS. The mean number of lesions in metastatic patients increased from 1.74 with CT to 1.88 with CEUS (p = 0.219 Wilcoxon's matched pairs signed rank test).

#### Comparison between dual-phase CT and conventional US

In 34/65 patients (52%), the number of lesions seen in conventional B-mode US and at dual-phase CT was identical. In the remaining 31 patients (47.7%) with a different number of lesions, more lesions were detected at dual-phase CT than at conventional B-mode US in 24 (37%) and more lesions at conventional B-mode US than at dual-phase CT in 7 (10.8%) patients. The mean number of lesions increased from 1.34 with conventional B-mode US to 1.74 with CT (p < 0.002 Wilcoxon's matched pairs signed rank test).

## Discussion

The present study shows that real time low mechanical index SonoVue-enhanced US and dual-phase helical CT are considerably more sensitive than baseline US in the detection of liver metastases from gastrointestinal cancers. Similar data have been reported for liver metastases in general, regardless of the primary site [17,25], and were, herewith, demonstrated, for the first time, selectively, in gastrointestinal cancers. From a clinical point of view, the present results are very important, since an accurate staging may significantly change the therapeutic approach in patients with liver metastases from gastrointestinal cancers.

Early and accurate detection of metastatic lesions in the liver, or exclusion of liver involvement, is a prerequisite for rational staging and follow-up of patients presenting cancers with the potential risk of developing metastases in the liver, as the first dissemination site. This is particularly true for colorectal cancers, in which metastases can be efficiently approached with surgical resection [26-29]. Optimal management implies scrupulous assessment of the feasibility of surgery, since patients with undetected metastases left *in situ *derive no, or dubious, therapeutic benefit from resection, indeed they risk complications and poor quality of life, and might benefit more from other treatment procedures.

Despite the fact that multidetector CT has been recommended as the reference method to stage colorectal cancer and diagnose liver metastasis [30], as well as or to monitor patients for recurrent colorectal cancer [31], conventional B-mode US is usually the first-step imaging technique in the assessment of metastatic liver disease in common clinical practice in various Italian institutions. Dual-phase CT and MRI are often reserved for patients in whom US has failed to show any lesions or is inconclusive or in those in whom thorough assessment of chest and abdomen is needed to plan subsequent treatment. Similar approaches can be found in other European Countries, such as The Netherlands, UK and France, as mentioned in the recent literature [28,32,33]. In the latter studies, reflecting clinical practice, the diagnosis of liver metastasis was established by definite US features alone, or by CT or by a combination of two. Furthermore, CT was recommended, in guidelines published in year 2000, only in the presence of clinical suspicion of possible recurrence [34]. Usually, no further assessment of the liver is required if US detects disseminated liver involvement [35]. The approach used in the present study seems, therefore, to well reflect current clinical practice in many European centers.

Accuracy of US, in the assessment of liver metastases, is lower than that of dual-phase CT and MRI, with reported values of 70–85% (76.9% in our series) in comparison to reference imaging [15,36-38] and far less than gold standards, including intra-operative exploration [11]. False-negative findings occur in as many as 15% of US studies. Despite these shortcomings, US offers numerous advantages: it is rapid, relatively inexpensive, easily available and free from ionizing radiation. Disadvantages of US include: the operator-dependent nature, difficulty related to the detection of isoechoic hepatic lesions and those <1 cm in size, as well as limitations in the characterization of hepatic lesions, which are not always metastatic, even in cancer patients [14,15]. CEUS may overcome some of the limitations of conventional B-mode, maintaining most of the advantages of conventional US.

The overall sensitivity of CEUS, in the detection of liver metastases, was comparable to that of spiral CT (Table 1) [23,25], but several metastatic lesions were detected by only one of the two techniques, as found when comparing CT with MRI [39] or other acquisition protocols with CT [8]. The burden of liver disease was not understaged, in any of the patients, by CEUS mode compared with conventional B mode. This is likely due to the sequential use of conventional US and CEUS, with CEUS being focused on all lesions detected at US, as well as, on the remaining parenchyma. The fact that a few new lesions were not detected at dual-phase CT, but were identified by CEUS, is in keeping with previous reports [18,24,25] and may be due to various reasons. Real-time modality, which allows prolonged continuous exploration of the liver (2–3 minutes in the late phase), the high spatial resolution of US with second harmonic imaging and the possibility to repeat the procedure more than once, in the same session, in the event of dubious lesions or difficult examinations, may all contribute to the identification of small or subcapsular lesions, which could be missed by CT, especially if not performed in specialized centers and with reliable equipment.

Assessment of metastatic lesions at the time of exam reporting and not on digitally recorded images, can be seen as a potential limitation of the study. However, it should not be forgotten that in most European Countries, US examinations are performed directly by physicians, who judge the presence of metastatic lesions during dynamic visualization of the liver. Liver studies are performed according to the needs and findings of each patient and do not follow thoroughly standardized protocols. Therefore, the present US study protocol reflects the routine approach in the clinical setting, although this differs from that of CT, for which assessment of metastatic lesions by multiple readers, blinded to each other, would have represented a more accurate study modality.

Another limitation of the study is the inclusion of multiple and different CT scanners. The use of a single type of top class equipment, with a larger number of detectors would probably have reduced the number of false negative results of CT in comparison to US modalities, but would have referred to conditions often different from everyday clinical practice, which the study protocol attempted to resemble as closely as possible.

Since the present data confirm, also for gastrointestinal cancers, the results of previous studies on the detection of liver metastases from neoplasms of various origin [23,25], CEUS of the liver could be recommended as an alternative to CT or MRI in cases presenting controindications for these techniques. Additionally CEUS may be used in association with CT, MRI or CT/PET in patients in whom failure to achieve accurate staging could result in not effecting adequate treatment or in whom most reliable prognostic findings are highly desireable [39-41].

Agreement has not yet been reached, concerning the validity and effectiveness of follow-up after primary gastrointestinal cancer resection or metastasectomy [42]. However, the improvement in the results of liver surgery, combined with chemotherapy and possibly with other treatment modalities, such as radiofrequency ablation, suggests that a follow-up program with sensitive imaging techniques, leading to the early detection of recurrences, may theoretically result in further improvement in the survival rate [43]. In the post-treatment follow-up setting, choice of which liver imaging technique should be performed remains a matter of debate, since, once again, costs, exposure to radiations and availability of resources need to be taken into account, all of which are much greater than when performed, only once, at staging. To date, there is no reliable evidence, that a surveillance program, with imaging modalities, is cost effective in colorectal cancer patients [42]. Some oncologic guidelines recommend post-treatment CT only in the presence of clinical suspicion of recurrent disease [34], but in the current practice this behavior varies considerably. Some oncologists follow these guidelines, whereas others recommend conventional US once every 6 months and others add, or perform only CT every 1 or 2 years [42]. Limitations to a more frequent use of CT, despite being considered a more accurate technique, from a mere imaging view-point [30], derive from costs, hazards related to ionizing radiations and to the use of iodine contrast agents as well as limited availability of resources in the follow-up setting. For these reasons, therefore, MRI and PET are poor candidates for periodic follow-up of cancer patients. CEUS, on the other hand, shows significantly better accuracy than conventional B-mode US, while presenting several of the advantages of US, namely: a) no of exposure to ionizing radiations; b) limited hazards related to the use of contrast agents, the severe adverse events ratio being extremely low [44]; c) low cost, even with the use of contrast agents; d) possibility of acquiring additional information from preliminary conventional B-mode US; e) theoretical widespread availability, since it can be installed in most US instruments. This new technique, therefore, appears to be an ideal candidate in the follow-up of patients not presenting liver metastases or following liver resection for primary gastrointestinal cancer, at least of colorectal origin. Randomized prospective studies are needed to confirm these findings.

## Conclusion

In conclusion CEUS is more sensitive than conventional US in the detection of liver metastases and could be usefully employed in the staging of patients with gastrointestinal cancer. Findings with CEUS and helical CT, with 1–4 detectors, appear comparable for overall sensitivity, but are not exactly superimposable in the identification of individual metastases. Thus, the two techniques could be considered complementary in those cases in which highest global sensitivity is required. Additionally, CEUS appears to have the potential to become the reference technique, in daily practice, for the early detection of metastastatic liver disease, at least in all those instances in which, at present, only an US scan is prescribed to monitor gastrointestinal cancer patients, without known liver involvement.

## Abbreviations

CEUS, Contrast Enhanced Ultrasound; CT, Computed Tomography; MRI, Magnetic Resonance Imaging; PET, Positron Emission Tomography; US, Ultrasound

## Competing interests

The authors declare that they have no competing interest.

Fabio Piscaglia, Francesco Giangregorio and Luigi Bolondi have received economical support from Bracco for expenses related to the attendance of scientific meetings (registration fees, travel and accomodation)

## Authors' contributions

FP conceived and coordinated the study, performed contrast US scans and revised the manuscript; FC collaborated in the collection of radiologic data and compilation of the database and drafted the initial manuscript; MM performed contrast US ultrasound scans; FG collaborated in collection of radiologic data and compilation of the database and performed contrast US scans; ST contributed to the design of the study and to patient recruitment; GU contributed to patient recruitment and helped in drafting the manuscript; BC contributed to patient recruitment; AB, PP and RR collaborated in collection of imaging reports, compilation of database and compilation of references; FF assisted in the coordination of the study and in collecting radiologic material; LB coordinated the study design and critically revised the manuscript. All authors read and approved the final manuscript.

## Pre-publication history

The pre-publication history for this paper can be accessed here:



## References

[B1] Paulson EK, McDermott VG, Keogan MT, DeLong DM, Frederick MG, Nelson RC (1998). Carcinoid metastases to the liver: role of triple-phase helical CT. Radiology.

[B2] Parker SL, Tong T, Bolden S, Wingo PA (1997). Cancer statistics, 1997. CA Cancer J Clin.

[B3] Porte R, Clavien P-A, C P-A (1999). Epidemiology and natural history of liver tumors. Malignant liver tumors: current and emerging therapies.

[B4] Wagner JS, Adson MA, Van Heerden JA, Adson MH, Ilstrup DM (1984). The natural history of hepatic metastases from colorectal cancer. A comparison with resective treatment. Ann Surg.

[B5] Cady B, Jenkins RL, Steele GD, Lewis WD, Stone MD, McDermott WV, Jessup JM, Bothe A, Lalor P, Lovett EJ (1998). Surgical margin in hepatic resection for colorectal metastasis: a critical and improvable determinant of outcome. Ann Surg.

[B6] Gazelle GS, Hunink MG, Kuntz KM, McMahon PM, Halpern EF, Beinfeld M, Lester JS, Tanabe KK, Weinstein MC (2003). Cost-effectiveness of hepatic metastasectomy in patients with metastatic colorectal carcinoma: a state-transition Monte Carlo decision analysis. Ann Surg.

[B7] Robinson P (2000). Imaging liver metastases: current limitations and future prospects. Br J Radiol.

[B8] Abdelmoumene A, Chevallier P, Chalaron M, Schneider F, Verdun FR, Frascarolo P, Meuli R, Schnyder P, Denys A (2005). Detection of liver metastases under 2 cm: comparison of different acquisition protocols in four row multidetector-CT (MDCT). Eur Radiol.

[B9] Staren ED, Gambla M, Deziel DJ, Velasco J, Saclarides TJ, Millikan K, Doolas A (1997). Intraoperative ultrasound in the management of liver neoplasms. Am Surg.

[B10] Clouse ME (1989). Current diagnostic imaging modalities of the liver. Surg Clin North Am.

[B11] Leen E, Ceccotti P, Moug SJ, Glen P, MacQuarrie J, Angerson WJ, Albrecht T, Hohmann J, Oldenburg A, Ritz JP (2006). Potential value of contrast-enhanced intraoperative ultrasonography during partial hepatectomy for metastases: an essential investigation before resection?. Ann Surg.

[B12] Ozarda A, Pickren J (1962). The topographic distribution of liver metastases its relation to surgical and isotope diagnosis. J Nucl Med.

[B13] Finlay IG, Meek D, Brunton F, McArdle CS (1988). Growth rate of hepatic metastases in colorectal carcinoma. Br J Surg.

[B14] Bleuzen A, Huang C, Olar M, Tchuenbou J, Tranquart F (2006). Diagnostic accuracy of contrast-enhanced ultrasound in focal lesions of the liver using cadence contrast pulse sequencing. Ultraschall Med.

[B15] Albrecht T, Hohmann J, Oldenburg A, Skrok J, Wolf KJ (2004). Detection and characterisation of liver metastases. Eur Radiol.

[B16] Dalla Palma L, Bertolotto M, Quaia E, Locatelli M (1999). Detection of liver metastases with pulse inversion harmonic imaging: preliminary results. Eur Radiol.

[B17] Quaia E, D'Onofrio M, Palumbo A, Rossi S, Bruni S, Cova M (2006). Comparison of contrast-enhanced ultrasonography versus baseline ultrasound and contrast-enhanced computed tomography in metastatic disease of the liver: diagnostic performance and confidence. Eur Radiol.

[B18] Quaia E, Bertolotto M, Forgacs B, Rimondini A, Locatelli M, Mucelli RP (2003). Detection of liver metastases by pulse inversion harmonic imaging during Levovist late phase: comparison with conventional ultrasound and helical CT in 160 patients. Eur Radiol.

[B19] Valls C, Lopez E, Guma A, Gil M, Sanchez A, Andia E, Serra J, Moreno V, Figueras J (1998). Helical CT versus CT arterial portography in the detection of hepatic metastasis of colorectal carcinoma. AJR Am J Roentgenol.

[B20] Strotzer M, Gmeinwieser J, Schmidt J, Fellner C, Seitz J, Albrich H, Zirngibl H, Feuerbach S (1997). Diagnosis of liver metastases from colorectal adenocarcinoma. Comparison of spiral-CTAP combined with intravenous contrast-enhanced spiral-CT and SPIO-enhanced MR combined with plain MR imaging. Acta Radiol.

[B21] Kinkel K, Lu Y, Both M, Warren RS, Thoeni RF (2002). Detection of hepatic metastases from cancers of the gastrointestinal tract by using noninvasive imaging methods (US, CT, MR imaging, PET): a meta-analysis. Radiology.

[B22] Albrecht T, Blomley M, Bolondi L, Claudon M, Correas JM, Cosgrove D, Greiner L, Jager K, de Jong N, Leen E (2004). Guidelines for the use of contrast agents in ultrasound. Ultraschall Med.

[B23] Albrecht T, Hoffmann CW, Schmitz SA, Schettler S, Overberg A, Germer CT, Wolf KJ (2001). Phase-inversion sonography during the liver-specific late phase of contrast enhancement: improved detection of liver metastases. AJR Am J Roentgenol.

[B24] Piscaglia F, Gaiani S, Tamberi S, Celli N, Cecilioni L, Gramantieri L, Bolondi L (2003). Liver metastases from rectal carcinoma: disease progression during chemotherapy despite loss of arterial-phase hypervascularity on real-time contrast-enhanced harmonic sonography at low acoustic energy. J Clin Ultrasound.

[B25] Oldenburg A, Hohmann J, Foert E, Skrok J, Hoffmann CW, Frericks B, Wolf KJ, Albrecht T (2005). Detection of hepatic metastases with low MI real time contrast enhanced sonography and SonoVue. Ultraschall Med.

[B26] Manfredi S, Lepage C, Hatem C, Coatmeur O, Faivre J, Bouvier AM (2006). Epidemiology and management of liver metastases from colorectal cancer. Ann Surg.

[B27] Leporrier J, Maurel J, Chiche L, Bara S, Segol P, Launoy G (2006). A population-based study of the incidence, management and prognosis of hepatic metastases from colorectal cancer. Br J Surg.

[B28] Silvera L, Galula G, Tiret E, Louvet C, Leroux JL, Trutt B (2006). Assessment of management practices for colonic cancer in the Paris metropolitan area in 2002. Gastroenterol Clin Biol.

[B29] Biasco G, Derenzini E, Grazi G, Ercolani G, Ravaioli M, Pantaleo MA, Brandi G (2006). Treatment of hepatic metastases from colorectal cancer: many doubts, some certainties. Cancer Treat Rev.

[B30] Schima W, Kulinna C, Langenberger H, Ba-Ssalamah A (2005). Liver metastases of colorectal cancer: US, CT or MR?. Cancer Imaging.

[B31] Faria SC, Tamm EP, Varavithya V, Phongkitkarun S, Kaur H, Szklaruk J, Dubrow R, Charnsangavej C (2005). Systematic approach to the analysis of cross-sectional imaging for surveillance of recurrent colorectal cancer. Eur J Radiol.

[B32] Bipat S, van Leeuwen MS, Ijzermans JN, Bossuyt PM, Greve JW, Stoker J (2006). Imaging and treatment of patients with colorectal liver metastases in The Netherlands: a survey. Neth J Med.

[B33] Griffiths EA, Browell DA, Cunliffe WJ (2005). Evaluation of a pre-operative staging protocol in the management of colorectal carcinoma. Colorectal Dis.

[B34] Benson Ar, Choti M, Cohen A, Doroshow J, Fuchs C, Kiel K, Martin EJ, McGinn C, Petrelli N, Posey J (2000). NCCN Practice Guidelines for Colorectal Cancer. Oncology (Williston Park).

[B35] Menu Y (2003). Liver metastases of colorectal cancers. Detection and delineation of their extension using imaging. Bull Acad Natl Med.

[B36] Carter R, Hemingway D, Cooke TG, Pickard R, Poon FW, McKillop JA, McArdle CS (1996). A prospective study of six methods for detection of hepatic colorectal metastases. Ann R Coll Surg Engl.

[B37] Hagspiel KD, Neidl KF, Eichenberger AC, Weder W, Marincek B (1995). Detection of liver metastases: comparison of superparamagnetic iron oxide-enhanced and unenhanced MR imaging at 1.5 T with dynamic CT, intraoperative US, and percutaneous US. Radiology.

[B38] Harvey CJ, Blomley MJ, Eckersley RJ, Cosgrove DO, Patel N, Heckemann RA, Butler-Barnes J (2000). Hepatic malignancies: improved detection with pulse-inversion US in late phase of enhancement with SH U 508A-early experience. Radiology.

[B39] Kim YK, Ko SW, Hwang SB, Kim CS, Yu HC (2006). Detection and characterization of liver metastases: 16-slice multidetector computed tomography versus superparamagnetic iron oxide-enhanced magnetic resonance imaging. Eur Radiol.

[B40] Bartolozzi C, Donati F, Cioni D, Procacci C, Morana G, Chiesa A, Grazioli L, Cittadini G, Giovagnoni A, Gandini G (2004). Detection of colorectal liver metastases: a prospective multicenter trial comparing unenhanced MRI, MnDPDP-enhanced MRI, and spiral CT. Eur Radiol.

[B41] Selzner M, Hany TF, Wildbrett P, McCormack L, Kadry Z, Clavien PA (2004). Does the novel PET/CT imaging modality impact on the treatment of patients with metastatic colorectal cancer of the liver?. Ann Surg.

[B42] Giovagnoni A, Ottaviani L, Mensa A, Durastanti M, Floriani I, Cascinu S (2005). Evidence Based Medicine (EBM) and Evidence Based Radiology (EBR) in the follow-up of the patients after surgery for lung and colon-rectal carcinoma. Radiol Med.

[B43] Ohlsson B, Palsson B (2003). Follow-up after colorectal cancer surgery. Acta Oncol.

[B44] Piscaglia F, Bolondi L, SIUMB Study Group on SonoVue (2006). The safety of SonoVue in abdominal applications. Retrospective analysis of 23.188 investigations. Ultrasound Med Biol.

